# Diabetic Retinopathy Diagnosis based on Convolutional Neural Network in the Russian Population: A Multicenter Prospective Study

**DOI:** 10.2174/0115733998268034231101091236

**Published:** 2023-11-28

**Authors:** Daria Gognieva, Madina Durzhinskaya, Irina Vorobyeva, Petr Chomakhidze, Alexander Suvorov, Natalia Kuznetsova, Alina Bektimirova, Baraah Al-Dwa, Magomed Abdullaev, Yusef Yusef, Vladislav Pavlov, Maria Budzinskaya, Dmitry Sychev, Larisa Moshetova, Philipp Kopylov

**Affiliations:** 1 World-Class Research Center “Digital Biodesign and Personalized Healthcare”, I.M. Sechenov First Moscow State Medical University (Sechenov University), Moscow, Russia;; 2 Research Institute of Eye Diseases Named After M.M. Krasnov, Federal State Budgetary Institution, Moscow, Russia;; 3 Federal State Budgetary Educational Institution of Further Professional Education “Russian Medical Academy of Continuous Professional Education” of the Ministry of Healthcare of the Russian Federation, Moscow, Russia

**Keywords:** Diabetes mellitus, diabetic retinopathy, diagnostics, screening, neural networks, machine learning

## Abstract

**Background:**

Diabetic retinopathy is the most common complication of diabetes mellitus and is one of the l*eading causes* of vision impairment globally, which is also relevant for the Russian Federation.

**Objective:**

To evaluate the diagnostic efficiency of a convolutional neural network trained for the detection of diabetic retinopathy and estimation of its severity in fundus images of the Russian population.

**Methods:**

In this cross-sectional multicenter study, the training data set was obtained from an open source and relabeled by a group of independent retina specialists; the sample size was 60,000 eyes. The test sample was recruited prospectively, 1186 fundus photographs of 593 patients were collected. The reference standard was the result of independent grading of the diabetic retinopathy stage by ophthalmologists.

**Results:**

Sensitivity and specificity were 95.0% (95% CI; 90.8-96.4) and 96.8% (95% CI; 95.5-99.0), respectively; positive predictive value – 98.8% (95% CI; 97.6-99.2); negative predictive value – 87.1% (95% CI, 83.4-96.5); accuracy – 95.9% (95% CI; 93.3-97.1); Kappa score – 0.887 (95% CI; 0.839-0.946); F1score – 0.909 (95% CI; 0.870-0.957); area under the ROC-curve – 95.9% (95% CI; 93.3-97.1). There was no statistically significant difference in diagnostic accuracy between the group with isolated diabetic retinopathy and those with hypertensive retinopathy as a concomitant diagnosis.

**Conclusion:**

The method for diagnosing DR presented in this article has shown its high accuracy, which is consistent with the existing world analogues, however, this method should prove its clinical efficiency in large multicenter multinational controlled randomized studies, in which the reference diagnostic method would be unified and less subjective than an ophthalmologist.

## 1 INTRODUCTION

According to the International Diabetes Federation reports in 2021, 537 million adults worldwide suffer from diabetes mellitus (DM). At the same time, it is expected that this figure will increase to 643 million by 2030, and to 783 million by 2045. In 2021, the estimated total mortality rate of DM was 6,700,000 [1]. In Russia, according to the Federal Register of Patients with Diabetes Mellitus, in 2021, DM was diagnosed in 4,799,552 people (3.23% of the country's population) [2]. In 2022, 345,000 new cases of the disease were detected [3]. The prevalence of diabetic retinopathy (DR) is 30 to 40% [4]. At the same time, according to the World Health Organization (WHO), DR ranks third among the causes of blindness in the world [5], which is also relevant for the Russian Federation [6].

In the United States of America, DM is the leading cause of blindness among the working adult population aged 20-74 years [7].

According to the order of the Ministry of Health of Russia, preventive examinations of apparently healthy adults should be carried out once every three years for people from 18 to 39 years old and annually for people over 40 years old. Routine ophthalmological examination with ophthalmoscopy is not included in the healthcare survey, but the evaluation of blood glucose levels in order to exclude glucose metabolism disorders is mandatory. It should also be noted that such examinations are often voluntary, and in case of the absence of symptoms of the disease, such as vision impairment and other manifestations of DM, a fairly large population group remains out of sight of the doctor and does not receive timely medical care.

If we consider the population group in which DM is already diagnosed, preventive fundus examination should be carried out annually. In case of absence of DR or mild and moderate DR, funduscopy should take place 2-3 times a year, in case of severe or proliferative DR - at least 3-4 times a year. However, the implementation of such a planned dynamic observation in practice is not always feasible, because patients suffering from DM may also have other complications (nephropathy, diabetic foot, *etc*.), which aggravate the general condition and may cause immobility. In addition, one cannot ignore the fact that the number of qualified specialists who are able to accurately diagnose retinal pathology is not always enough, especially in remote regions, which also limits access to qualified medical care.

Thus, there is wide clinical and research potential for developing a portable, non-invasive, cost-effective screening and diagnostic tool for DR with high diagnostic efficiency and reproducibility. Neural networks, which have already proven to be highly effective in DR detection, can become such a tool.

This article presents the results of a multicenter prospective study aimed at evaluating the effectiveness of DR detection and grading using a convolutional neural network (CNN) developed by a team of scientists from Russia. This study is the first and currently the only one in the Russian Federation.

## 2 MATERIALS AND METHODS

This cross-sectional multicenter study was conducted according to the principles of the Helsinki Declaration and was approved by the Local Ethics Committee of Sechenov University (protocol number 05-21, March 10, 2021). For the purpose of neural network training, a public dataset was used (https://www.kaggle.com/c/diabetic-retinopathy-detection/data). Digital fundus photographs from this source were independently relabeled twice by seven certified ophthalmologists with at least 2 years of professional experience in retinology in order to ensure high accuracy in diagnosing macular pathology. All images were reduced to a resolution of 1024x1024 px, their color was normalized to average values according to the “dataset”. The labeled sample included 30,000 patients (60,000 eyes) and it was divided into two parts: 80% – training dataset, and 20% validation set. The basic CNN was trained in three steps:

1. Training of the first six layers of the CNN on 224x224 px image resolution.
2. The use of nine layers with 448x448 px image resolution.
3. Full CNN training with 1024x1024 px image resolution.

This technique enables the gradient descent during the error backpropagation at the early stages of training, which improves the convergence and generalization of the network. A further increase in image resolution does not lead to an improvement in classification accuracy. To obtain an expert CNN, all layers of the base CNN, except for the last ones, were frozen and only fully connected layers were trained using the training dataset. At each training step, the neural network traversed the same dataset 200 times (epochs) or until the training error on the validation data set stopped decreasing. The architecture of the neural network is presented in Table **1**. All codes used in the study were executed in the TensorFlow 2.0 environment with Windows 10 + CUDA (Compute Unified Device Architecture) 10.0. TensorFlow is an open source machine learning library developed by Google® for building and training neural networks. The training was conducted on a hardware platform with a 3.60 GHz Intel Core i9-9900K processor with 32.0 GB of RAM and an NVIDIA GeForceRTX 2080 graphics card.

### 2.1 First Stage

The inclusion of patient data in the test sample was carried out according to the previously calculated sample size on the basis of the Cardiology Department of the University Clinical Hospital No.1. of Sechenov University (237 patients, 474 eyes) and the Department of retinal and optic nerve pathology of the Federal State Budgetary Institution “Research Institute of Eye Diseases named after M.M. Krasnov” (356 people, 712 eyes). This study included 350 patients with a confirmed DM (according to data from medical documentation, which is stored in the electronic database of the clinics), of which 310, in addition to DM and diabetic retinopathy, had a confirmed diagnosis of arterial hypertension and also signs of hypertensive retinopathy; the control group consisted of 242 people, of which 156 had cardiac diagnosis and the corresponding hypertensive retinopathy, and 86 people did not have DM or arterial hypertension and any macular pathology. After inclusion in the study, all patients were assigned a unique identification number for the purpose of blinding, further work was carried out with depersonalized data. Images were obtained using a medical digital camera “Optomed Smartscope PRO”, Optomed USA Inc. San Francisco, CA. Fundus photography was performed under the condition of adequate pharmacological mydriasis with the usage of the drug “Fenikamide” (international non-proprietary name tropicamide + phenylephrine, manufacturer GROTEX, LLC, reg. No.: LP-004830 dated 04/26/2018). After receiving 8-field fundus photographs (according to Early Treatment of Diabetic Retinopathy Study (ETDRS) protocol requirements), a number identical to the unique patient's number was assigned to the photographs, and then all of the photos were sent to the specific cloud storage. Subsequent image analysis was performed by two independent ophthalmologists with at least 2 years of professional experience in retinology. Ambiguous clinical cases, that required the adoption of a collegial expert decision, were resolved. Based on the analysis, ophthalmologists graded the photographs according to image quality (high, medium, low, very poor – cannot be interpreted), patients whose photographs were of very poor quality were excluded from further analysis at this step. After that, confirmation or refutation of the diagnosis of DR and identification of the stage of DR were carried out based on International Council of Ophthalmology (ICO) Guidelines for Diabetic Eye Care, 2017 (Table **2**) [8].

An average time required for the analysis of one photo by each specialist was calculated.

At this stage, due to poor image quality, 53 people were excluded from the study (30 with a diagnosis of arterial hypertension, 21 with combination of DM and arterial hypertension, and 2 healthy volunteers).

### 2.2 Second Stage

Along with the fundus photography, the researchers conducted analysis of medical documentation from the electronic database, followed by creating specific database containing metadata of the study sample. The presence of the diagnosis “DM” was recorded when one or more of the following WHO criteria were met: Fasting venous or capillary plasma glucose ≥ 7.0 mmol/L (126 mg/dL), 2-hour post-load venous plasma glucose ≥ 11.1 mmol/L (200 mg/dL), 2-hour post-load capillary plasma glucose ≥ 12.2 mmol/L (220 mg/dL), HbA1c 6.5% (48 mmol/mol) [9]. In addition, the presence of aggravations in the form of hypertension, coronary heart disease, atrial fibrillation, ventricular arrhythmias and severe renal pathology was recorded.

### 2.3 Third Stage

At this stage, the test sample was passed through the neural network. To obtain predictions, a CNN was used, it more detailed description can be found in paper of Tatarkanov A., *et al.* [10]. The main principle of this process is extraction of important simple features of an object from an image (borders, points, color transitions, *etc*.) and filtering out all unimportant ones. This results in a generation of a single number, indicating that the sample belongs to some certain classification section.

It should be noted that due to the large amount of data (8-field fundus photographs per one eye), in order to reduce resource costs and unify the technique, it was decided to use only the photo of the central field for analysis. All photographic images used at this stage for testing the CNN were subjected to pre-processing, the resolution was unified to 1024x1024 *px*, and the color was normalized to average values ​​according to the dataset.

After completion of testing, confirmation of the presence or absence of the diagnosis of DR and determination of its stage was obtained for all of the fundus photographs. The time required for analysis of each image by CNN was fixed. Analysis could not be performed for some images due to poor quality at this stage of the study, therefor, 1 patient was excluded.

## 3 EXPERIMENTAL

Point estimation and the creation of graphs were carried out using the Python v.3.10 programming language, based on the scikit-learn v.1.2 module. The bootstrap method was used to calculate the 95% confidence interval. Continuous variables with normal distribution were presented as mean (standard deviation); non-normal variables were reported as median (interquartile range). *P*-values < 0.05 were considered statistically significant. The diagnosis of DR, made by independent ophthalmologists, was used as a reference standard. The efficiency of the CNN has been estimated in terms sensitivity, specificity, positive and negative predictive value, accuracy, Cohens Kappa score, F1 score, and area under Receiver Operating Characteristic curve (ROC analysis). These parameters were also determined in the classification of DR by stages.

## 4 RESULTS

The final sample consisted of 540 people (1080 eyes), 299 (55.37%) of which were female. The mean age was 56±14.08 years. Researchers reached different conclusions when analyzing fundus photographs of the test sample in 21 cases, the final decision on each specific case was made collectively, with the involvement of a third independent ophthalmologist.

**Fig. (1):**
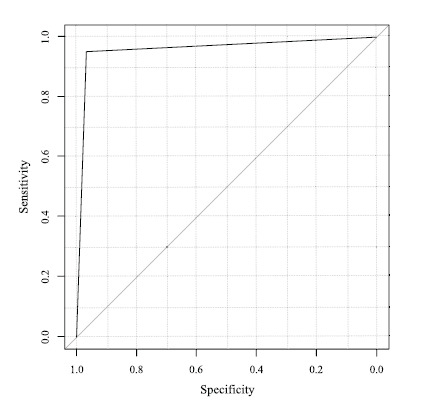
Area under Receiver Operating Characteristic curve for convolutional neural network in DR detection.

Sensitivity and specificity in DR detection were 95.0% (95% CI; 90.8-96.4) and 96.8% (95% CI; 95.5-99.0), respectively; positive predictive value – 98.8% (95% CI; 97.6-99.2); negative predictive value – 87.1% (95% CI, 83.4-96.5); accuracy (accuracy) – 95.9% (95% CI; 93.3-97.1); Cohens Kappa score – 0.887 (95% CI; 0.839-0.946); F1score – 0.909 (95% CI; 0.870-0.957); area under the ROC-curve – 95.9% (95% CI; 93.3-97.1) (Fig. **1**).

Table **3** presents the indicators of the diagnostic efficiency of the developed neural network for various stages of DR.

Fig. (**2**) shows the area under the ROC curve for each stage of DR.

Determination of diagnostic efficiency was also performed separately for patients with DR (n=40) and with combination of diabetic and hypertensive retinopathy (n=310). There was no statistically significant difference in diagnostic efficiency for these groups.

The average time for labeling one fundus image for an ophthalmologist was 1 minute 14 seconds, while for a neural network, this figure was 3 minutes, of which the processing itself took 0.8 milliseconds.

## 5 DISCUSSION

The first study dedicated to an application of neural network for the screening of DR was published by Gardner *et al*. about 30 years ago [11]. Scientific progress, accompanied not only by the improvement of optical equipment and digitalization necessary to create high-quality databases of big data of medical images, but also by an increase in the efficiency of neural networks themselves, has led to an increase in interest in research aimed at DR screening using neural networks in recent years.

In the first work of Gardner *et al*. from 1996 the sensitivity and specificity of back propagation neural network in diabetic retinopathy detection was 88.4% and 83.5%, respectively [9]. At the same time, current neural networks demonstrate higher diagnostic accuracy, with sensitivity and specificity greater than 90% [12]. According to a systematic review and meta-analysis by Mohaimenul *et al*., which included 20 articles published from January 2000 to March 2019, the sensitivity of methods based on machine learning was 0.83 (95% CI: 0.83-0.83), the specificity was 0.92 (95% CI: 0.92-0.92), and the area under the ROC curve – 0.97 (95% CI: 0.95-0.98). At the same time, the authors noted that the methodology of the reviewed studies was quite heterogeneous, which may affect the result, and in most of them, the diagnosis established by an ophthalmologist was used as a reference, which does not allow to exclude a possible component of subjective diagnostic error [13].

In our study, the mean sensitivity was 95.0% (95% CI; 90.8-96.4), specificity was 96.8% (95% CI; 95.5-99.0), and area under the ROC curve was 95.9% (95% CI; 93.3-97.1), which is generally consistent with the results described in the world literature.

The study by Choi *et al*. revealed that the diagnostic efficiency of the neural network decreased in cases of various corresponding fundus pathologies (combination of DR with hypertensive retinopathy, age-related macular degeneration, *etc.*) [14]. In this regard, patients in our study were divided into two groups: Those who had only one fundus pathology – DR and those who, in addition to DR, also had hypertensive retinopathy. As mentioned above, the presence of signs of hypertensive retinopathy did not have a significant impact on the indicators of diagnostic efficiency.

It should be noted that in the course of data analysis, ophthalmologists considered fundus photographs of 53 patients unsuitable for analysis due to its low quality, while the CNN was unable to analyze the data of only 1 patient. Unfortunately, we were unable to evaluate the diagnostic capabilities of the CNN on the specified set of poor quality images, because the diagnosis established by an ophthalmologist was used as a reference in our study.

As for the disagreements that arose between ophthalmologists regarding the diagnosis in 21 cases, in 13 (61.9%) of them, the diagnosis made by CNN coincided with the reference, a discrepancy was noted in 8 (38.1%) cases. All of the above indicates that, unlike a person, a machine is not characterized by doubts when making decisions, fatigue and professional burnout, which undoubtedly creates prerequisites for the use of such diagnostic models for mass screening in conditions of limited material and health human resources. At the same time, researchers do not yet have an understanding of exactly what processes take place within the so-called “black box”, which implies the emergence of organizational, legal and ethical problems with widespread introduction into practical healthcare. In the study conducted by Lynch *et al*. fundus photographs containing typical signs of DR were amended at the pixel level, invisible to the human eye. Further, these photographs were passed through the neural network, which had previously been trained with a large amount of images containing signs of the indicated pathology, and it did not reveal pathological changes and attributed them as “normal”. At the same time, a neural network trained to diagnose certain pathological changes (lesion-based approach) in the fundus made the diagnosis correctly [15].

## 6 LIMITATIONS

Despite the rather high diagnostic efficiency obtained in the course of our study, it has certain limitations: Firstly, a public database of digital fundus photographs, which does not contain any additional information about patients, including race and nationality was used for the training and validation of the neural network, which theoretically could influence the results; secondly, the inclusion of patients data in the test sample was carried out in Moscow clinics and ethnicity data was not taken into account, which does not allow to expand the results on the multinational population of Russia (represented by more than 160 nationalities); thirdly, the neural network used in our study was trained using images and is also characterized by the “black box” phenomenon and the possible resulting problems described above.

## CONCLUSION

The method for DR detection presented in this article has shown its high efficiency, which is generally consistent with the results of world analogues. To date, the evidence base for the use of neural networks for the detection and screening of DR seems to be quite impressive. However, it becomes obvious that for a wider clinical application, these methods should prove their effectiveness in large multicenter multinational randomized trials in which the reference diagnostic method would be unified and less subjective than an ophthalmologist.

## Figures and Tables

**Fig. (2):**
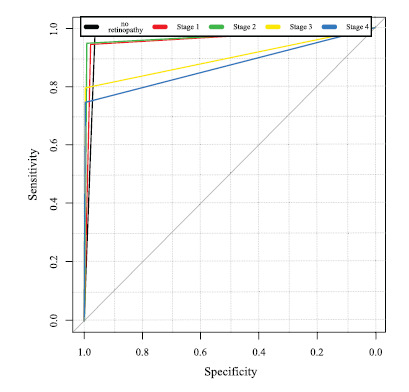
Area under the ROC curve for each stage of DR.

**Table 1:** Neural network architecture.

**Name**	**Units**	**Filter**	**Stride**	**Size**
1 Input	-	-	-	448
2 Conv	32	5	2	224
3 Conv	32	3	-	224
4 MaxPool	-	3	2	111
5 Conv	64	5	2	56
6 Conv	64	3	56	-
7 Conv	64	3	56	-
8 MaxPool	3	-	2	27
9 Conv	128	3	-	27
10 Conv	128	3	-	27
11 Conv	128	3	-	27
12 MaxPool	-	3	2	13
13 Conv	256	3	-	13
14 Conv	256	3	-	13
15 Conv	256	3	-	13
16 MaxPool	3	-	2	6
17 Conv	512	3	-	6
18 Conv	512	3	-	6
19 MaxPool	3	-	2	6
20 Conv	1024	3	-	6
21 Conv	1024	3	-	6
22 RMSPool	-	3	3	2
23 Dropout	-	-	-	-
24 Dense	1024	-	-	-
25 Maxout	512	-	-	-
26 Dropout	-	-	-	-
27 Dense	1024	-	-	-
28 Maxout	512	-	-	-

**Table 2:** International classification of diabetic retinopathy.

**Disease Findings**	**Observable on Dilated Ophthalmoscopy**
No apparent DR	No abnormalities
Mild nonproliferative DR	Microaneurysms only
Moderate nonproliferative DR	Microaneurysms and other signs (*e.g.,* dot and blot hemorrhages, hard exudates, cotton wool spots), but less than severe nonproliferative DR
Severe nonproliferative DR	Moderate nonproliferative DR with any of the following: intraretinal hemorrhages (≥20 in each quadrant); definite venous beading (in 2 quadrants); intraretinal microvascular abnormalities (in 1 quadrant); and no signs of proliferative retinopathy
Proliferative DR	Severe nonproliferative DR and 1 or more of the following: neovascularization, vitreous/preretinal hemorrhage

**Note:** DR – diabetic retinopathy.

**Table 3:** Diagnostic efficiency of the convolutional neural network for various stages of DR.

**Diagnostic Efficiency Indicators**	**Stage 1**	**Stage 2**	**Stage 3**	**Stage 4**
AUC	96.5 (95% CI; 80.9 – 98.9)	97.2 (95% CI; 94.2-98.4)	89.8 (95% CI; 81.7-98.7)	87.4 (95% CI; 80.6-98.5)
Sensitivity, %	95.0 (95% CI; 89.6- 100.0)	95.2 (95% CI; 88.8-97.2)	80.0 (95% CI; 63.7-97.7)	75.0 (95% CI; 61.5-97.1)
Specificity, %	98.0 (95% CI; 96.7- 98.5)	99.1 (95% CI; 98.9-99.7)	99.6 (95% CI; 99.2-99.9)	99.8 (95% CI; 99.62-100.0)
NPV	79.1 (95% CI; 68.9- 84.1)	90.9 (95% CI; 87.9-97.9)	80.0 (95% CI; 68.0-97.7)	85.7 (95% CI; 75.6-100.0)
PPV	99.5 (95% CI; 99.2- 100.0)	99.5 (95% CI; 99.0-99.8)	99.6 (95% CI; 99.0-99.9)	99.6 (95% CI; 99.0-99.9)
F1	0.863 (95% CI; 0.795- 0.896)	0.930 (95% CI; 0.900-0.972)	0.800 (95% CI; 0.727-0.894)	0.800 (95% CI; 0.691-0.975)
Balanced accuracy, %	96.4 (95% CI; 93.7- 98.9)	97.2 (95% CI; 94.2-98.4)	89.8 (95% CI; 81.7-98.7)	79.7 (95% CI; 68.6-97.4)
Kappa score	0.851 (95% CI; 0.777- 0.886)	0.924 (95% CI; 0.892-0.970)	0.796 (95% CI; 0.721-0.892)	0.797 (95% CI; 0.686-0.974)
Weighted kappa	0.895 [0.846, 0.943]			

**Abbreviations:** AUC – area under the curve; CI – confidence interval; NPV – negative predictive value; PPV – positive predictive value

## Data Availability

The data that support the findings of this study are available from the corresponding author, [D.G.], on special request.

## LIST OF ABBREVIATIONS

AUC: Area Under the Curve
CI: Confidence Interval
CNN: Convolutional Neural Network
CUDA: Compute Unified Device Architecture
DM: Diabetes Mellitus
DR: Diabetic Retinopathy
ETDRS: Early Treatment of Diabetic Retinopathy Study
GAP: Global Average Pooling
ICO: International Council of Ophthalmology
NPV: Negative Predictive Value
PPV: Positive Predictive Value
RAM: Random Access Memory
WHO: World Health Organization

## STANDARDS OF REPORTING

STROBE guidelines were followed.

## References

[1] (2021). IDF Diabetes Atlas. Diabetes around the world in. https://diabetesatlas.org/.

[2] Dedov II, Shestakova MV, Vikulova OK, Zheleznyakova AV, Isakov MA (2021). Epidemiological characteristics of diabetes mellitus in the Russian Federation: clinical and statistical analysis according to the Federal diabetes register data of 01.01.2021.. Diabetes mellitus.

[3] (2022). TASS. 345 000 new patients with diabetes were identified in Russia in. https://tass.ru/obschestvo/16313061.

[4] Tan T.E., Wong T.Y. (2023). Diabetic retinopathy: Looking forward to 2030.. Front. Endocrinol. (Lausanne).

[5] World health organization Blindness and vision impairment.. https://www.who.int/news-room/fact-sheets/detail/blindness-and-visual-impairment.

[6] Ministry of Health of the Russian Federation Report of the chief ophthalmologist of the Ministry of Health of the Russian Federation.. https://minzdrav.gov.ru/news/2022/10/13/19398-glavnyy-vneshtatnyy-oftalmolog.

[7] Center for disease control and prevention. Common eye disorders and diseases.. https://www.cdc.gov/visionhealth/basics/ced/index.html#:~:text=The%20leading%20causes%20of%20blindness,disorders%20include%20amblyopia%20and%20strabismus.

[8] Wong T.Y., Sun J., Kawasaki R. (2018). Guidelines on diabetic eye care.. Ophthalmology.

[9] Diagnosis and management of type 2 diabetes (HEARTS-D). Geneva: World Health Organization 2020. (WHO/UCN/NCD/20.1). Licence: CC BY-NC-SA 3.0 IGO.

[10] Tatarkanov A., Alexandrov I., Glashev R. (2021). Synthesis of neural network structure for the analysis of complex structured ocular fundus images.. J Appl Eng Sci.

[11] Gardner G.G., Keating D., Williamson T.H., Elliott A.T. (1996). Automatic detection of diabetic retinopathy using an artificial neural network: a screening tool.. Br. J. Ophthalmol..

[12] Ahmad B.U., Kim J.E., Rahimy E. (2020). Fundamentals of artificial intelligence for ophthalmologists.. Curr. Opin. Ophthalmol..

[13] Islam M.M., Yang H.C., Poly T.N., Jian W.S., Jack Li Y.C. (2020). Deep learning algorithms for detection of diabetic retinopathy in retinal fundus photographs: A systematic review and meta-analysis.. Comput. Methods Programs Biomed..

[14] Choi J.Y., Yoo T.K., Seo J.G., Kwak J., Um T.T., Rim T.H. (2017). Multi-categorical deep learning neural network to classify retinal images: A pilot study employing small database.. PLoS One.

[15] Lynch S.K., Shah A., Folk J.C., Wu X., Abramoff M.D. (2017). Catastrophic failure in image-based convolutional neural network algorithms for detecting diabetic retinopathy.. Invest. Ophthalmol. Vis. Sci..

